# An open-label randomized clinical trial of prophylactic paracetamol coadministered with 7-valent pneumococcal conjugate vaccine and hexavalent diphtheria toxoid, tetanus toxoid, 3-component acellular pertussis, hepatitis B, inactivated poliovirus, and *Haemophilus influenzae* type b vaccine

**DOI:** 10.1186/1471-2431-13-98

**Published:** 2013-06-21

**Authors:** Markus A Rose, Christine Juergens, Beate Schmoele-Thoma, William C Gruber, Sherryl Baker, Stefan Zielen

**Affiliations:** 1 Department of Paediatric Pulmonology/Allergy/Infectious Diseases, Children’s Hospital, Goethe University, Theodor Stern Kai 7, Frankfurt/Main, 60590, Germany; 2 Pfizer Pharma GmbH, Berlin, Germany; 3 Pfizer Vaccine Research, Pearl River, NY, USA; 4 Former employee, Pfizer Vaccine Research, Pearl River, NY, USA

**Keywords:** Fever, Pneumococcal conjugate vaccine, Hexavalent vaccine, Paracetamol, Prophylaxis

## Abstract

**Background:**

In two clinical trials, low-grade fever was observed more frequently after coadministration than after separate administration of two recommended routine pediatric vaccines. Since fever is an important issue with vaccine tolerability, we performed this open-label study on the efficacy and safety of prophylactic use of paracetamol (acetaminophen, Benuron®) in children administered routine 7-valent pneumococcal conjugate vaccine (PCV-7) coadministered with hexavalent vaccine (diphtheria-tetanus-acellular pertussis-hepatitis B, poliovirus, *Haemophilus influenzae* type b vaccine [DTPa-HBV-IPV/Hib]) in Germany.

**Methods:**

Healthy infants (N = 301) who received a 3-dose infant series of PCV-7 and DTPa-HBV-IPV/Hib plus a toddler dose were randomly assigned 1:1 to prophylactic paracetamol (125 mg or 250 mg suppositories, based on body weight) at vaccination, and at 6–8 hour intervals thereafter, or a control group that received no paracetamol. Rectal temperature and local and other systemic reactions were measured for 4 days post vaccination; adverse events were collected throughout the study.

**Results:**

In the intent-to-treat population, paracetamol reduced the incidence of fever ≥38°C, but this reduction was only significant for the infant series, with computed efficacy of 43.0% (95% confidence interval [CI]: 17.4, 61.2), and not significant after the toddler dose (efficacy 15.9%; 95% CI: −19.9, 41.3); results were similar in the per protocol (PP) population. Fever >39°C was rare during the infant series, such that there were too few cases for assessment. After the toddler dose, paracetamol effectively reduced fever >39°C, reaching statistical significance in the PP population only (efficacy 79%; 95% CI: 3.9, 97.7). Paracetamol also reduced reactogenicity, but there were few significant differences between groups after any dose. No vaccine-related serious adverse events were reported.

**Conclusions:**

Paracetamol effectively prevented fever and other reactions, mainly during the infant series. However, as events were generally mild and of no concern in either group our data support current recommendations to administer paracetamol to treat symptoms only and not for routine prophylaxis.

**Trial registration:**

NCT00294294

## Background

In two clinical trials, infants who received 7-valent pneumococcal conjugate vaccine (PCV-7) concomitantly with a hexavalent vaccine (diphtheria, tetanus, acellular pertussis, hepatitis B, inactivated poliovirus, *Haemophilus influenzae* type b [DTPa-HBV-IPV/Hib]) had an almost 2-fold higher incidence of febrile reactions than those who received these combination vaccines alone [1,2]. Since febrile reactions play a major role in the parents’ perception of vaccine tolerability and might even stop parents from having their infants immunized, some health care providers tend to prophylactically administer paracetamol (acetaminophen, Benuron®) as part of immunization practice. Data on this preventative strategy are scarce; therefore, to assess this approach, we performed a study on the effect of prophylactic paracetamol administration on febrile and other common vaccine reactions following routine coadministration of PCV-7 and DTPa-HBV-IPV/Hib.

## Methods

### Study design and participants

This was an open-label, randomized study conducted at 22 pediatric offices in Germany. The study was approved by the ethics committee of the Goethe-Universität, Germany, and conducted from May 2005 to December 2006 in accordance with the Declaration of Helsinki. Written informed consent was obtained from parents/legal guardians from all subjects before enrollment. Healthy infants aged 56–112 days were enrolled in the study. Contraindications to study participation were a gestational age <37 weeks and/or a birth weight <2,500 g and/or a body weight <4,000 g at enrollment; failure to thrive; immune deficiency or suppression; severe congenital malformations or neurological or other severe chronic disorders; any history of seizures; bleeding disorders; prior administration of blood products, other vaccines, or antipyretics for other indications; a known intolerance to paracetamol or the scheduled vaccines; or participation in another investigational study.

### Interventions

Subjects received PCV-7 (Prevenar®, Pfizer Inc; containing pneumococcal serotypes 4, 6B, 9V, 14, 18C, 19F, and 23F) and DTPa-HBV-IPV/Hib (INFANRIX hexa™, GlaxoSmithKline) at ages 2, 3, and 4 months (infant series) and at age 11–14 months (toddler dose) as 0.5 mL intramuscular injections into the left and right anterolateral thigh, respectively. The prophylaxis group received three paracetamol suppositories at 6–8 hour intervals, receiving the first immediately after vaccination. Children weighing <7 kg received paracetamol 375 mg/day; children weighing 7 to <10 kg received paracetamol 500 mg/day; and children weighing ≥10 kg received paracetamol 750 mg/day (given as 125mg and 250mg suppositories). If required for therapeutic purposes, paracetamol was to be offered to the participants at the investigator’s discretion. To prevent overdose, subjects in the prophylaxis group were not to receive paracetamol in addition to their study medication on the day of vaccination or sooner than 6 hours after their last dose.

### Assessments

In an electronic diary, the parents or legal guardians recorded the subject’s core (rectal) temperature using a digital thermometer in the evening of the day of vaccination (day 1) and in the morning and evening of days 2–4. Fever was defined as the endogenous elevation of at least one measured body temperature of ≥38°C [3].

Local reactions were measured using a caliper on a numeric scale from 1 to 14 (or 14+ if larger), and systemic reactions were recorded in the e-diary on days 1–4 along with the time and dose of paracetamol treatment, and any other non-study antipyretic treatment. Adverse events (AEs) were recorded by the investigator on the case report form at each visit.

### Statistical methods

The sample size was based on the following assumptions: the incidence of fever following any of the first three doses in the control group (without antipyretic prophylaxis) was 65%; ≥50% reduction in incidence of fever; ≤15% of subjects would drop out before the end of the infant series; with alpha = 0.05, 2-sided. A total of 120 evaluable subjects per group provided ≥95% power to show a lower 95% confidence interval (CI) >0 for the incidence of fever following any of the first three doses. For the toddler dose, assumptions were as follows: ≥70% of subjects completing the infant series returned for their toddler dose; the incidence of fever following the toddler dose in the control group was 40%; ≥50% reduction in incidence of fever was desired; with alpha = 0.10, 2-sided. A total of 90 evaluable subjects per group provided ≥80% power to show a lower 90% CI >0 for the incidence of fever. To ensure an overall sufficient sample size, 150 subjects per group were to be enrolled.

Participants were randomly assigned in a 1:1 ratio to the prophylaxis group or to the control group using the sponsor’s Clinical Operations Randomization Environment II system with a block size of 4. Subjects who discontinued after random assignment were not replaced.

### Populations for analysis

The intent-to-treat (ITT) population included any randomly assigned subject, with or without fever ≥38°C who had at least 1 recorded post vaccination temperature. However, a decision to include subjects in the actual ITT analysis of fever (denominator N) was dependent on whether or not data was missing. To be counted as not having a fever, all temperature measurements were required. Thus, if a subject’s highest temperature was 38.5°C and had at least 1 missing temperature measurement, then this subject would be included in the analysis of fever ≥ 38°C and excluded from the analysis of fever >39°C. Inclusion of subjects with missing data as “absent” (no fever) would otherwise lower the rate of fever reported.

The per protocol (PP) population included subjects who received their allocated medication, all four doses of study vaccine, and had a sufficient number of temperatures recorded to permit evaluation. For handling missing data the same logic as for the ITT population was applied. The safety population included all subjects who received at least one dose of study vaccine. Subjects who lacked any safety data (AE, reactogenicity, or temperature) for a particular vaccination were excluded from that analysis. Separate safety populations were defined for each vaccination.

The primary endpoint was the incidence of fever (core temperature ≥38°C) after the infant series and after the toddler dose in each group. The secondary endpoint was the incidence of fever >39°C after each dose in each group. The incidence of fever in the prophylaxis group relative to the control group (relative risk [RR]) and 2-sided 95% CI estimates of efficacy (1−RR) were evaluated. The 2-sided, 95% CIs were computed using exact methods, conditional upon the total number of subjects reporting fever. If there were fewer than five subjects with fever in the control group, then only the estimate of efficacy was computed and no CIs were presented.

The safety endpoints included the incidence of local and systemic reactions, and AEs after each vaccination. AEs were categorized according to the Medical Dictionary for Regulatory Activities. Comparisons between groups were performed using a 2-sided Fisher exact test.

## Results

**Figure 1:**
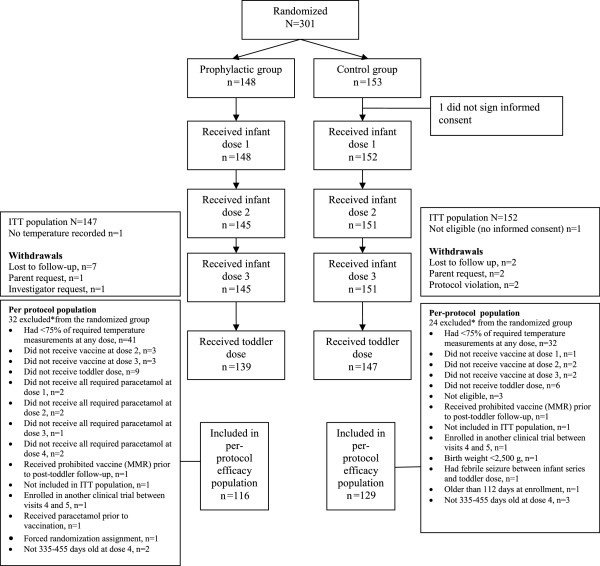
Flow diagram of the trial *Withdrawals from the intent-to-treat (ITT) population are included among withdrawals from the per protocol (PP) population; subjects may be excluded for more than one reason. MMR = measles, mumps and rubella.

A total of 301 subjects were randomly assigned to the prophylaxis group (n = 148) or to the control group (n = 153). Details of the ITT and PP populations are presented in Figure 1. The demographic characteristics of the ITT population were generally similar across groups except that there were slightly more males in the control group (Table 1); the demographic characteristics of the PP and safety populations were similar to those of the ITT population (data not shown).

**Table 1:** **Demographic characteristics: intent**-**to**-**treat efficacy population**

		**Prophylaxis group**	**Control group**	**Total**
		**n = 147**	**n = 152**	**N = 299**
**Gender**	Female	50.3%	46.7%	48.5%
	Male	49.7%	53.3%	51.5%
**Race**	White	98.6%	98%	98.3%
	Asian	0.7%	0.7%	0.7%
	Black / other	0.7% / 0%	0.7% / 0.7%	0.7% / 0.3%
Body weight* **in kg, median (min, max)**		5.9 (4.0, 7.6)	5.9 (4.4, 8.2)	5.9 (4.0, 8.2)
**Age (months), median (min, max)**	Dose 1	2.4 (1.9, 3.7)	2.4 (1.9, 3.8)	2.4 (1.9, 3.8)
	Dose 2	3.6 (2.8, 5.0)	3.7 (2.7, 5.2)	3.6 (2.7, 5.2)
	Dose 3	4.7 (3.8, 6.4)	4.8 (3.8, 6.3)	4.7 (3.8, 6.4)
	Toddler dose	11.7 (10.2, 15.8)	11.6 (11.0, 16.8)	11.7 (10.2, 16.8)

* Weight at enrollment.

### Efficacy of antipyretic prophylaxis

In the ITT analysis population, the incidence of fever ≥38°C during the infant series was significantly reduced in the prophylaxis group compared with the control group; however, the toddler dose of paracetamol had no significant impact on fever ≥38°C (Table 2). Similar results were observed in the PP analysis population (Table 2). Fever >39°C after the infant series was an overall rare event, and was observed in ≤5 subjects, limiting the assessment of paracetamol efficacy in both the ITT and PP analyses populations. After the toddler dose, paracetamol’s prevention of fever >39°C reached significance in the PP analysis population only (Table 2).

**Table 2:** Efficacy of antipyretic prophylaxis in preventing fever within 4 days of vaccination

**ITT analysis population**				**PP analysis population**		
	**Prophylaxis group**	**Control group**	Efficacy*	**Prophylaxis group**	**Control group**	Efficacy*
	**% (n/N)**	**% (n/N)**	**% (95% CI)**	**% (n/N)**	**% (n/N)**	**% (95% CI)**
**Fever ≥38°C**				**Fever ≥38°C**		
**Infant series**	43.0 (43/100)	75.4 (95/126)	43.0 (17.4, 61.2)	36.3 (29/80)	75.9 (82/108)	52.3 (26.3, 69.9)
**Toddler dose**	53.7 (58/108)	63.9 (76/119)	15.9 (−19.9, 41.3)	51.6 (49/95)	61.5 (67/109)	16.1 (−23.1, 43.2)
**Fever >39°C**				**Fever >39°C**		
**Dose 1**	0	4.0 (5/124)	100.0 (−17.7, 100)	0	4.6 (5/109)	100.0 (−22.6, 100.0)
**Dose 2**	0	1.8 (2/112)	100.0 (NA)	0	2.0 (2/100)	100.0 (NA)
**Dose 3**	1.0 (1/102)	1.9 (2/103)	49.5 (NA)	1.1 (1/87)	1.1 (1/91)	−4.6 (NA)
**Toddler dose**	4.6 (4/87)	13.1 (13/99)	65.0 (−13.3, 91.7)	2.6 (2/78)	12.2 (11/90)	79.0 (3.9, 97.7)

*Efficacy is 1 − relative risk (prophylaxis group relative to control group). Confidence interval (CI) is computed using exact methods conditional upon the number of subjects having reported the specific fever. No CI was computed when <5 subjects reported a febrile reaction. Differences between groups is significant if lower limit of 95% CI >1 for computed efficacy and similar if 95% CI includes 1. *ITT* intent-to-treat population, *PP* per-protocol population, *N* number of subjects included in the analysis, *n* number of subjects with the specified degree of fever, *NA* not applicable.

The percentage of subjects administered therapeutic non-study antipyretics in the ITT population within 15 days post vaccination was higher in the control group than in the prophylaxis group for both the infant series (35.5% and 19.6%, respectively) and after the toddler dose (32.0% and 17.3%, respectively).

### Local reactions after PCV-7 and DTPa-HBV-IPV/Hib

The majority of local reactions were mild or moderate in severity. The incidence of local reactions increased after the toddler dose. Local reactions were less frequent in the prophylaxis group; however, statistical significance was only reached after PCV-7 for tenderness after dose 1 (p = 0.049) and for redness after dose 2 (p = 0.017) (Table 3).

**Table 3:** Local reactions at the PCV-7 and DTPa-HBV-IPV/Hib injection site within 4 days of vaccination – safety population

		**PCV-7**			**DTPa-HBV-IPV/Hib**		
**Local reactions**		**Prophylaxis group% (n/N)**	**Control group% (n/N)**	p-value*	**Prophylaxis group% (n/N)**	**Control group% (n/N)**	p-value*
**Tenderness**^†^	Dose 1	5.3 (6/114)	12.9 (17/132)	0.049	6.1 (7/114)	11.5 (15/130)	0.180
	Dose 2	8.3 (10/120)	14.4 (17/118)	0.157	10.0 (12/120)	15.4 (18/117)	0.244
	Dose 3	5.5 (6/109)	10.3 (11/107)	0.216	6.4 (7/109)	10.2 (11/108)	0.337
	Toddler dose	21.3 (20/94)	32.7 (35/107)	0.082	21.2 (21/99)	29.9 (32/107)	0.202
	Toddler dose/severe	3.4 (3/88)	5.1 (5/98)	0.724	3.4 (3/88)	3.1 (3/98)	>0.99
**Swelling**^‡^	Dose 1	9.5 (11/116)	17.2 (23/134)	0.096	17.9 (21/117)	18.2 (24/132)	>0.99
	Dose 2	17.1 (21/123)	27.2 (34/125)	0.067	23.4 (29/124)	34.6 (44/127)	0.053
	Dose 3	16.8 (18/107)	27.7 (31/112)	0.074	21.8 (24/110)	28.1 (32/114)	0.355
	Toddler dose	24.2 (23/95)	33.3 (35/105)	0.164	37.3 (38/102)	37.5 (39/104)	>0.99
**Redness**^‡^	Dose 1	22.5 (27/120)	27.2 (37/136)	0.470	20.8 (25/120)	23.7 (31/131)	0.650
	Dose 1/severe	0.9 (1/114)	0	0.475	0.9 (1/114)	0	0.475
	Dose 2	21.1 (26/123)	34.9 (44/126)	0.017	30.4 (38/125)	40.6 (52/128)	0.115
	Dose 3	25.4 (29/114)	34.8 (39/112)	0.147	28.9 (33/114)	34.2 (39/114)	0.476
	Toddler dose	34.7 (35/101)	44.4 (48/108)	0.160	44.2 (46/104)	48.1 (51/106)	0.583

*Fisher exact test, 2-sided.

^†^ Severe tenderness was defined as present and interfered with limb movement.

^‡^ Severe swelling and redness was defined as an area >7 cm.

*n* number of subjects reporting the event, *N* number of subjects reporting “yes” for at least 1 day or “no” for all days, *NA* not applicable.

### Systemic reactions

Systemic reactions were generally less frequent in the prophylaxis group with significant differences noted for fever, rash, irritability, drowsiness, decreased appetite, persistent inconsolable crying, and decreased activity after at least one dose in the infant series (all p < 0.05). After the toddler dose, where systemic reactions occurred more often, only “activity” was significantly decreased in the prophylaxis group (p = 0.005) (Table 4).

**Table 4:** Systemic events within 4 days of PCV-7 and DTPa-HBV-IPV/Hib vaccination –safety population

**Systemic event**	**Dose**	**Prophylaxis group % (n/N)**	**Control group % (n/N)**	p-value*
**Fever**	Dose 1	9.3 (11/118)	35.8 (48/134)	<0.001
**≥38°C to ≤39°C**	Dose 2	19.7 (24/122)	43.7 (55/126)	0.000
	Dose 3	19.3 (21/109)	45.6 (52/114)	0.000
	Toddler dose	51.5 (53/103)	60.0 (69/115)	0.221
**>39°C to ≤40°C**	Dose 1	0	4.0 (5/124)	0.061
	Dose 2	0	1.8 (2/112)	0.238
	Dose 3	1.0 (1/102)	1.9 (2/103)	>0.99
	Toddler dose	4.6 (4/87)	13.1 (13/99)	0.072
**>40°C**^**†**^	Toddler dose	0	1.1 (1/95)	>0.99
**Rash**	Dose 1	17.6 (21/119)	17.1 (22/129)	>0.99
	Dose 2	6.8 (8/118)	15.7 (19/121)	0.040
	Dose 3	12.7 (14/110)	22.4 (24/107)	0.074
	Toddler dose	13.3 (12/90)	23.6 (25/106)	0.098
**Irritability**	Dose 1	47.2 (59/125)	62.1 (87/140)	0.019
	Dose 2	42.2 (54/128)	58.5 (76/130)	0.013
	Dose 3	39.7 (48/121)	50.0 (59/118)	0.120
	Toddler dose	48.2 (53/110)	60.5 (75/124)	0.066
**Drowsiness**	Dose 1	50.4 (65/129)	64.7 (90/139)	0.019
	Dose 2	46.5 (59/127)	58.3 (74/127)	0.078
	Dose 3	36.4 (43/118)	45.6 (52/114)	0.182
	Toddler dose	43.5 (47/108)	50.4 (59/117)	0.350
**Decreased appetite**	Dose 1	30.3 (37/122)	40.0 (54/135)	0.118
	Dose 2	26.6 (33/124)	42.7 (53/124)	0.011
	Dose 3	23.0 (26/113)	33.6 (37/110)	0.101
	Toddler dose	38.2 (39/102)	45.2 (52/115)	0.336
**Persistent inconsolable crying**	Dose 1	9.5 (11/116)	20.0 (26/130)	0.031
	Dose 2	9.3 (11/118)	15.8 (19/120)	0.171
	Dose 3	14.0 (15/107)	15.3 (17/111)	0.849
	Toddler dose	7.8 (7/90)	17.1 (18/105)	0.056
**Decreased activity**	Dose 1	41.6 (52/125)	46.3 (63/136)	0.457
	Dose 2	31.0 (39/126)	48.0 (60/125)	0.007
	Dose 3	23.3 (27/116)	40.0 (46/115)	0.007
	Toddler dose	29.0 (29/100)	48.3 (56/116)	0.005

n = number of subjects experiencing the event. N = Number of subjects reporting “yes” for at least 1 day or “no” for all days.

*Fisher exact test, 2-sided.

^**†**^One severe event occurred after toddler dose only.

Seventeen serious adverse events (SAEs) were reported in 10 subjects (three subjects in the control group and seven in the prophylaxis group), and were medical conditions commonly seen in this age group. Two subjects in the prophylaxis group experienced seizures; one subject had an afebrile seizure on day 6 after the third dose of study vaccine, and one had febrile seizure on day 24 after the toddler dose. No SAEs were considered related to the study vaccines or study medication. No safety-related discontinuations or deaths were reported in this study.

## Discussion

This study showed that prophylactic paracetamol significantly prevented fever ≥38°C after routine administration of PCV-7 and DTPa-HBV-IPV/Hib when administered during the infant series, but not after the toddler dose. Fever >39°C was an overall rare event during the infant series, limiting a statistical assessment; however, after the toddler dose, paracetamol significantly prevented fever >39°C in the PP analysis population, but not in the ITT analysis population.

Due to the very conservative approach taken of handling missing data, the incidence of fever reported in this manuscript may be an over-estimate of the true rate. Inclusion of subjects with missing data would otherwise have lowered the rate of fever reported. Therefore, there is a potential of a bias, which may have caused a reduction in the assessment of efficacy of prophylactic paracetamol. True efficacy may be higher than what is reported here.

Paracetamol also tended to prevent local reactions at the injection sites, but for the majority there were no significant differences between groups, especially after the toddler dose. Paracetamol significantly reduced the incidence of all systemic reactions after at least one dose during the infant series; but again, there were no significant differences between groups after the toddler dose except for the assessment of decreased activity, which was observed less frequently in the paracetamol group.

Our data are generally consistent with that of other studies [4-7], but not with studies where a single dose of paracetamol was administered, which reported no significant impact on any reactions [8,9]. In all studies, paracetamol generally seemed to have less impact after the toddler dose. Two studies in children aged 4–6 years who received a fifth dose of DTPa, or a booster dose of diphtheria-tetanus-pertussis (whole cell) vaccine, reported no significant impact of paracetamol on the incidence of local reactions [5,10]. Local reactions in children generally occur more frequently after a booster dose such that the weak anti-inflammatory mechanism of paracetamol may not be sufficient to control inflammation, suggesting that ibuprofen may be a better alternative if required [11].

Of note, recent studies with pneumococcal conjugate vaccines and DTPa-HBV-IPV/Hib have shown that prophylactic paracetamol reduced immunogenicity of all antigens studied. However, a high proportion of participants still achieved immune responses believed to correlate with protection, suggesting that vaccine efficacy was not impacted [6,12].

Overall, fever reactions were mainly mild and of little concern to parents. Fever >39°C was rarely observed after the infant series and occurred in approximately 13% of the control group after the toddler dose. However, exposure of a whole population to prophylactic antipyretics to prevent fever in the minority does not seem justified, whereas targeted treatment of symptoms would reduce the number of individuals unnecessarily exposed to the risk of toxicity [13].

The major limitation of this study was the lack of immunogenicity data; this is currently being addressed in a study assessing the prophylactic use of ibuprofen and paracetamol on immunogenicity of 13-valent pneumococcal conjugate vaccine when coadministered with DTPa-HBV-IPV/Hib [14]. The relatively higher dose of paracetamol used in this study to prevent fever was due to the lack of availability of suppositories in smaller doses at the time of the study, limiting weight-adjusted dosing.

## Conclusion

The data support current recommendations [15] that analgesic/antipyretics should be given only to treat clinically relevant post-vaccination symptoms and not for routine prophylaxis.

## Abbreviations

AEs: Adverse events; CI: Confidence interval; DTPa-HBV-IPV/Hib: Diphtheria Tetanus Acellular pertussis Hepatitis B Inactivated poliovirus *Haemophilus influenza* type b; ITT: Intent-to-treat; PCV-7: 7-valent pneumococcal conjugate vaccine; PP: Per-protocol; RR: Relative risk; SAEs: Serious AEs.

## Competing interests

MAR and SZ are consultants to Wyeth/Pfizer Inc and have received travel grants or honoraria within the past 3 years. Pfizer paid the article-processing charge for this article. CJ, BS-T, and WCG are employees of Pfizer Inc. SB was an employee of Wyeth, which was acquired by Pfizer Inc in October 2009.

## Authors’ contributions

All authors participated in the design, implementation, analysis, and interpretation of the study; the writing of the report; and the decision to submit for publication. SZ was the coordinating investigator, MAR led the clinical team. CJ, BS-T, and WCG managed the study at Wyeth, which was acquired by Pfizer Inc in October 2009. SB was the statistician for the study. All authors read and approved the final manuscript.

## Pre-publication history

The pre-publication history for this paper can be accessed here:

http://www.biomedcentral.com/1471-2431/13/98/prepub
